# mbDecoda: a debiased approach to compositional data analysis for microbiome surveys

**DOI:** 10.1093/bib/bbae205

**Published:** 2024-05-02

**Authors:** Yuxuan Zong, Hongyu Zhao, Tao Wang

**Affiliations:** Department of Bioinformatics and Biostatistics, Shanghai Jiao Tong University, Shanghai, China; SJTU-Yale Joint Center of Biostatistics and Data Science, Shanghai Jiao Tong University, Shanghai, China; SJTU-Yale Joint Center of Biostatistics and Data Science, Shanghai Jiao Tong University, Shanghai, China; Department of Biostatistics, Yale University, New Haven, CT; Department of Bioinformatics and Biostatistics, Shanghai Jiao Tong University, Shanghai, China; SJTU-Yale Joint Center of Biostatistics and Data Science, Shanghai Jiao Tong University, Shanghai, China; Department of Statistics, Shanghai Jiao Tong University, Shanghai, China

**Keywords:** ANCOM, Bias correction, Differential abundance testing, Zero-inflated models

## Abstract

Potentially pathogenic or probiotic microbes can be identified by comparing their abundance levels between healthy and diseased populations, or more broadly, by linking microbiome composition with clinical phenotypes or environmental factors. However, in microbiome studies, feature tables provide relative rather than absolute abundance of each feature in each sample, as the microbial loads of the samples and the ratios of sequencing depth to microbial load are both unknown and subject to considerable variation. Moreover, microbiome abundance data are count-valued, often over-dispersed and contain a substantial proportion of zeros. To carry out differential abundance analysis while addressing these challenges, we introduce mbDecoda, a model-based approach for debiased analysis of sparse compositions of microbiomes. mbDecoda employs a zero-inflated negative binomial model, linking mean abundance to the variable of interest through a log link function, and it accommodates the adjustment for confounding factors. To efficiently obtain maximum likelihood estimates of model parameters, an Expectation Maximization algorithm is developed. A minimum coverage interval approach is then proposed to rectify compositional bias, enabling accurate and reliable absolute abundance analysis. Through extensive simulation studies and analysis of real-world microbiome datasets, we demonstrate that mbDecoda compares favorably with state-of-the-art methods in terms of effectiveness, robustness and reproducibility.

## INTRODUCTION

High-throughput sequencing has emerged as a fundamental tool in microbiome research. Following careful experimental design and sample collection, raw sequence reads are generated through marker gene, metagenome or meta-transcriptome sequencing. Bioinformatics pipelines are then employed to quantify the abundances of numerous features, including operational taxonomic units, amplicon sequence variants or functional elements, ultimately yielding high-dimensional feature tables or matrices. Subsequent analyses are conducted on these feature tables to unveil overarching patterns in microbiome variation. For example, researchers often apply standard statistical tests to identify taxa that exhibit significant associations with variables of interest (for example, case control status or other health outcomes) [1]. However, it is important to note that the feature tables only provide relative, not absolute, abundance of each feature in each sample. Compositionality lies at the heart of the issue [2, 3]. When the proportion of one taxon increases, the proportions of others must decrease for the proportions to sum to one. For example, even if the treatment does not affect other taxa, their relative abundances would decrease due to the increase of a single taxon. This poses a challenge for using relative abundances to infer about absolute abundances, resulting in an increase in both false positives and false negatives [4].

Various normalization methods, such as Wrench [5] and GMPR [6], have been developed to address the challenge of comparing samples with varying sequencing depth. These methods are then combined with a model-based approach for conducting differential abundance analysis, with popular examples including DESeq2 [7], edgeR [8] and metagenomeSeq [9]. However, most of these methods are not efficient at mitigating the compositionality bias, because the microbial loads and sampling depths are both unknown and variable [10]. Here, in contrast to sequencing depth, which represents the amount of sequencing data generated for a single sample, sampling depth, also known as sampling fraction, is defined as the ratio of sequencing depth to total microbial load, assuming an equal probability of sequencing any microbial cell [11, 12]. Alternatively, several methods have been proposed that directly utilize relative abundances to make inferences about absolute abundances. For instance, ANCOM addresses compositionality by employing pairwise log-ratios among features [13]. Extending this approach, fastANCOM enhances computational efficiency and provides P-values for making inferences [14]. To handle zero values in the data, a common practice is to add a small positive constant before performing log transformations, although the choice of this constant is not made based on rigorous statistical criteria. Other strategies for addressing compositionality involve referencing a taxon or set of taxa that exhibit non-differential abundance between groups or lack associations with the variable of interest. Methods like LOCOM exemplify this approach [15–17]. However, these methods heavily rely on prior knowledge or valid inference of the reference. A novel class of methods, including ANCOMBC and LinDA, first obtain biased estimates and then adjust the test statistics by correcting for the compositionality bias [18, 19]. Specifically, ANCOMBC accounts for sampling depth by introducing a sample-specific offset term in a linear regression model for log-transformed observed abundances. This offset term is estimated for bias correction. LinDA assumes a linear regression model for log-transformed absolute abundances, and at the sample level, a bias term is introduced through the use of centered log-ratio transformation on observed abundances. This bias term is estimated and corrected. In addition to these statistical and computational methods that enhance the analysis of microbiome compositions, various experimental techniques have been developed, such as spike-in, flow cytometry, qPCR and dPCR [20–23]. Each of these techniques has its own limitations, which are beyond the scope of this paper.

In addition to compositionality, microbiome abundance data are count-valued, and over-dispersed with a substantial proportion of zeros. One way of dealing with over-dispersion is to use transformations, with logarithmic transformation being the most widely employed method [13, 14, 18, 19]. Historically, when dealing with discrete variables, there has been a tendency to transform them in a way that approximates a normal distribution with constant variance. This enables the application of classical statistical methods. However, in many practical cases, achieving this transformation is not feasible. In particular, to deal with zero values in the data, one needs to avoid logarithms of these zeros. As mentioned earlier, simply adding a pseudo count is a somewhat arbitrary approach. More sophisticated methods exist [19, 24], but their practical impact and statistical robustness necessitate thorough investigation. Alternatively, the theory and methodology of generalized linear models and their variants offer a way to handle data without the need for transformations to conform to normality. This model-based approach is simple and flexible, and it lends itself to easier interpretation compared with transformation-based approaches. To address sparsity and over-dispersion, common strategies in microbiome data analysis involve the use of zero-inflated Poisson or negative-binomial models [25, 26]. However, it is worth noting that most existing methods for differential abundance analysis, or more generally, association analysis, fail to account for compositionality [27, 28]. Recently, Hu *et al.* [15] introduced LOCOM, a logit model-based approach for compositional analysis of microbiome data that does not rely on pseudo counts. This represents a significant advancement, although the challenges related to data sparsity and over-dispersion remain largely unresolved.

**Figure 1:**
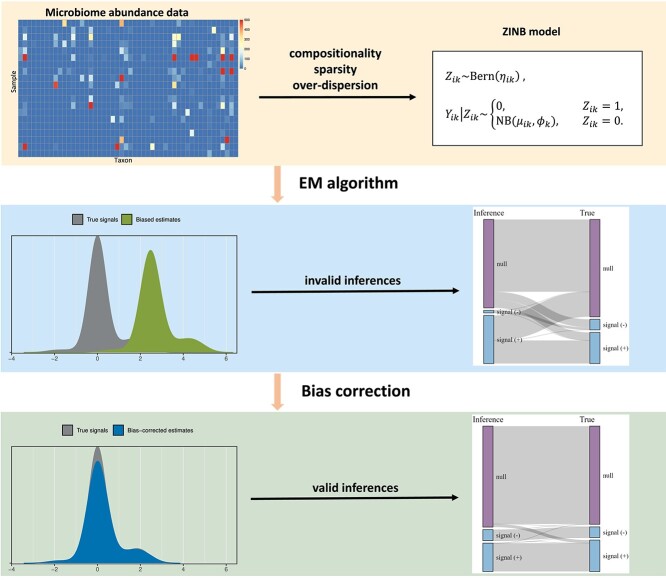
Schematic illustration of mbDecoda. mbDecoda utilizes the ZINB model to address challenges related to compositionality, sparsity and over-dispersion, thereby eliminating the need for data normalization and transformation (upper panel). Key components of mbDecoda include an EM algorithm for parameter estimation and an MCI strategy for mitigating compositional bias. The middle panel illustrates the bias present in estimates derived from the EM algorithm, which results in invalid inferences characterized by high false positive and false negative rates. The lower panel demonstrates that, following bias correction, valid inferences can be made with significantly improved true positive and true negative rates.

In this paper, we propose mbDecoda, a model-based approach to debiased compositional data analysis for microbiome surveys (Figure 1). To mitigate the issues of sparsity and overdispersion, mbDecoda describes microbiome abundance data using a zero-inflated negative binomial (ZINB) distribution. Furthermore, it relates the mean abundance to the variable of interest via the log link function and supports the adjustment for covariates or confounding factors. An efficient Expectation Maximization (EM) algorithm, which alternates between logistic regression and weighted NB regression in the M-step, is developed to approximately obtain the maximum likelihood estimates of model parameters. Note that, to account for unequal sampling depths, a sample-level term is incorporated in the same way as in ANCOMBC when modeling the association between the variable of interest and the abundance level of a taxon; please refer to Section 2.1 for detailed information on model specification. However, including this term renders the model parameters unidentifiable. Moreover, employing an ad-hoc normalization method to estimate this term does not resolve the identifiability issue but rather introduces uncertainty into estimates of model parameters. To address this challenge, after the estimation step, a minimum coverage interval (MCI) approach is then proposed to correct for the compositional bias. Importantly, this bias-correction step is separated from the estimation step, ensuring accurate and reliable absolute abundance analysis. Table 1 presents a concise summary of mbDecoda and representative absolute abundance analysis methods. The effectiveness and robustness of these methods are evaluated using simulated examples, with data generated from a wide range of models, and the application of these methods to real microbiome datasets is illustrated, with an emphasize on assessing their reproducibility across different datasets.

**Table 1:** Summary of strategies used by different absolute abundance analysis methods based on their treatment of three characteristics of microbiome abundance data: compositionality, sparsity and over-dispersion. The last column indicates whether each method provides a P-value

Method	Compositionality	Sparsity	Over-dispersion	P-value
ANCOM	Pairwise log-ratios	Pseudo-count	Transformation	No
fastANCOM	Pairwise log-ratios	Pseudo-count	Transformation	Yes
ANCOMBC	Bias correction	ANCOM-II	Transformation	Yes
LinDA	Bias correction	Pseudo-count/imputation	Transformation	Yes
LOCOM	Reference taxon	Logit model	No	Yes
mbDecoda	Bias correction	ZINB model	ZINB model	Yes

## METHODS

### Model specification

We define $i=1,\ldots ,n$ as the index of samples, and $k=1,\ldots ,K$ as the index of taxa. Let $Y=(Y_{i k})$ denote the observed $n\times K$ matrix of counts. To address data sparsity and over-dispersion, we consider a ZINB model of the form 


(2.1a) \begin{align*} {\label{1a}} Z_{i k} &\sim \mathrm{Bern}\left(\eta_{i k}\right), \end{align*}

(2.1b) \begin{align*} Y_{i k} \mid Z_{i k} &\sim \begin{cases} 0, & Z_{i k}=1,\\ \mathrm{NB} \left(\mu_{i k},\phi_{k}\right), &Z_{i k}=0, \end{cases} \end{align*}

where Bern denotes the Bernoulli distribution, $\eta _{i k}$ is the probability parameter of zero inflation and $\mu _{ik}$ and $\phi _{k}$ are the mean and dispersion parameters of the NB distribution. The latent variables $Z=\left (Z_{i k}\right )$ distinguish true absence (taxa that do not exist in the sample) from undetected presence (taxa that are not observed due to insufficient sequencing or low abundance).

Let $X=\left (X_{1}, \ldots ,X_{n}\right )^{\top }$ denote the observed vector of a variable of interest. To link microbial counts with this variable while accounting for possible confounders, we specify $\eta _{ik}$ and $\mu _{ik}$, respectively, as 


(2.1c) \begin{align*} & \mathrm{logit} \left(\eta_{i k}\right)=U_{i}^{\top} \alpha_{k}, \end{align*}

(2.1d) \begin{align*} & \label{1d}\log \left(\mu_{i k}\right)=d_{i}+X_{i}\Delta_{k}+V_{i}^{\top} \beta_{k}, \end{align*}

where $U_{i}=\left ( 1, U_{i1}, \ldots ,U_{ip_{1}}\right )^{\top }$ and $V_{i}=\left ( 1, V_{i1}, \ldots ,V_{ip_{2}}\right )^{\top }$ are vectors of covariates, and $\alpha _{k}=\left ( \alpha _{k0}, \alpha _{k1}, \ldots ,\alpha _{kp_{1}}\right )^{\top }$and $\beta _{k}=\left ( \beta _{k0}, \beta _{k1}, \ldots ,\beta _{kp_{2}}\right )^{\top }$ are the corresponding coefficients. Often, $U_{i}$ and $V_{i}$ are assumed to be the same. However, it is crucial to carefully select features that could potentially impact the differential abundance analysis as covariates. Furthermore, $\Delta _{k}$ represents the log fold change of the $k$-th taxon associated with each unit increase of $X_{i}$. Notably, the observed abundance cannot fully reflect the absolute abundance due to the absence of sampling depths [11, 18]. Therefore, the unknown sample-specific term $d_{i}$ in (2.1d) represents a compositional effect.

### Estimation and algorithm

Write $d=\left ( d_{1},\ldots ,d_{n}\right )^{\top }$, $\Delta =\left ( \Delta _{1},\ldots ,\Delta _{K}\right )^{\top }$, $\beta =\left ( \beta _{1}, \ldots ,\beta _{K}\right )^{\top }$, $\alpha =\left ( \alpha _{1}, \ldots ,\alpha _{K}\right )^{\top }$, and $\phi =\left ( \phi _{1}, \ldots ,\phi _{K}\right )^{\top }$. Let $\Theta =\{d, \Delta , \beta , \alpha , \phi \}$. We propose an EM algorithm to estimate these parameters. The complete data log-likelihood is expressed as 


\begin{align*}&\begin{aligned} l_{c} (\Theta \mid Y, Z)=& \sum_{i} \sum_{k} \left\{Z_{i k} \log \left(\eta_{i k}\right)+\left(1-Z_{i k}\right) \log \left(1-\eta_{i k}\right)\right\}\\ &+\sum_{i} \sum_{k} \left(1-Z_{i k}\right) \log p_{N B}\left(Y_{i k} \mid \mu_{i k}, \phi_{k}\right), \end{aligned}\end{align*}

where 


\begin{align*}& p_{N B}\left(Y_{i k} \mid \mu_{i k}, \phi_{k}\right)=\frac{\Gamma\left(Y_{i k}+\phi_{k}\right)}{\Gamma\left(\phi_{k}\right) Y_{i k} !}\left(\frac{\phi_{k}}{\mu_{i k}+\phi_{k}}\right)^{\phi_{k}}\left(\frac{\mu_{i k}}{\mu_{i k}+\phi_{k}}\right)^{Y_{i k}}. \end{align*}

Here, $\Gamma \left (\cdot \right )$ is the gamma function, and the symbol ‘ ! ’ denotes the factorial operation.

The EM algorithm iteratively refines the parameter estimates. In the $t$-th E-step, we calculate the expectation of $l_{c}(\Theta \mid Y, Z)$ with expect to $Z$ given $Y$ under the current estimate $\Theta ^{(t)}$. Let $\eta _{i k}^{(t)}=(1+\exp (-U_{i}^{\top } \alpha _{k}^{(t)}))^{-1}$ and $\mu _{i k}^{(t)}=\exp (d_{i}^{(t)}+X_{i}\Delta _{k}^{(t)}+V_{i}^{\top } \beta _{k}^{(t)})$. It is easy to show that 


\begin{align*}\begin{aligned} Z_{i k}^{(t)}\stackrel{\triangle} {=}E\left(Z_{i k} \mid Y_{i k}, \Theta^{(t)}\right)&= \begin{cases}\frac{\eta_{i k}{ }^{(t)}}{\eta_{i k}^{(t)}+\left(1-\eta_{i k}^{(t)}\right)\left(\frac{\phi_{k}^{(t)}}{\mu_{i k}^{(t)}+\phi_{k}^{(t)}}\right)^{\phi_{k}^{(t)}}}, &\quad \text{if}\ Y_{i k}=0, \\ 0, &\quad \text{if}\ Y_{i k}>0.\end{cases} \end{aligned} \end{align*}

Hence, 


\begin{align*}&\begin{aligned} Q\left(\Theta^{(t)}, \Theta\right)\stackrel{\triangle }=\,& E_{Z}\left\{ l_{c}(\Theta \mid Y, Z) \mid Y,\Theta^{(t)}\right\} \\ =& \sum_{i} \sum_{k} \left\{Z_{i k}^{(t)} \log \left(\eta_{i k}\right)+\left(1-Z_{i k}^{(t)}\right) \log \left(1-\eta_{i k}\right)\right\}\\ &+\sum_{i} \sum_{k} \left(1-Z_{i k}^{(t)}\right) \left[ \log \left\{\Gamma\left(Y_{i k}+\phi_{k}\right)\right\}+\phi_{k}\log\left(\phi_{k}\right)\right.\\& \ \ +Y_{i k}\log\left(\mu_{i k}\right) \\ & \left.-\log \left\{\Gamma\left(\phi_{k}\right)\right\}-\log\left(Y_{i k}!\right)-\phi_{k}\log\left(\mu_{ik}+\phi_{k}\right)\right.\\ &\ \ \ \ \left.-Y_{i k}\log\left(\mu_{i k}+\phi_{k}\right) \right]\\ =\,& Q_{1}\left(Z^{(t)},\alpha \right) +Q_{2}\left(Z^{(t)},d,\Delta, \beta,\phi \right). \end{aligned} \end{align*}

In the $t$-th M-step, we update $\Theta $ by maxmizing $Q\left (\Theta ^{(t)}, \Theta \right )$ with respect to $\Theta $, namely, 


\begin{align*}& \Theta^{(t+1)}=\arg \max_{\Theta} Q\left(\Theta^{(t)}, \Theta\right) . \end{align*}

Given the natural decomposition of the Q-function into two parts, we proceed to optimize the parameters of each part individually as follows: 


\begin{align*}& \alpha^{(t+1)}=\arg \max_{\alpha} Q_{1}\left(Z^{(t)}, \alpha\right), \end{align*}

\begin{align*}& \{d^{(t+1)},\Delta^{(t+1)}, \beta^{(t+1)},\phi^{(t+1)}\}=\arg \max_{\{d, \Delta, \beta, \phi\}}Q_{2}\left(Z^{(t)},d,\Delta,\beta,\phi \right). \end{align*}

A simple algebraic analysis shows that the form of $Q_{1}$ corresponds to the loss function of logistic regression, while $Q_{2}$ matches with the log-likelihood of weighted NB regression. Note that the parameter $d_{i}$ is specific to each sample, while $\Delta _{k}$, $\beta _{k}$ and $\phi _{k}$ vary across taxa. To estimate these parameters efficiently, we alternatively estimate them by treating one set as fixed while maximizing $Q_{2}$ with respect to the other set. The procedure of parameter estimation is summarized in Algorithm 1.


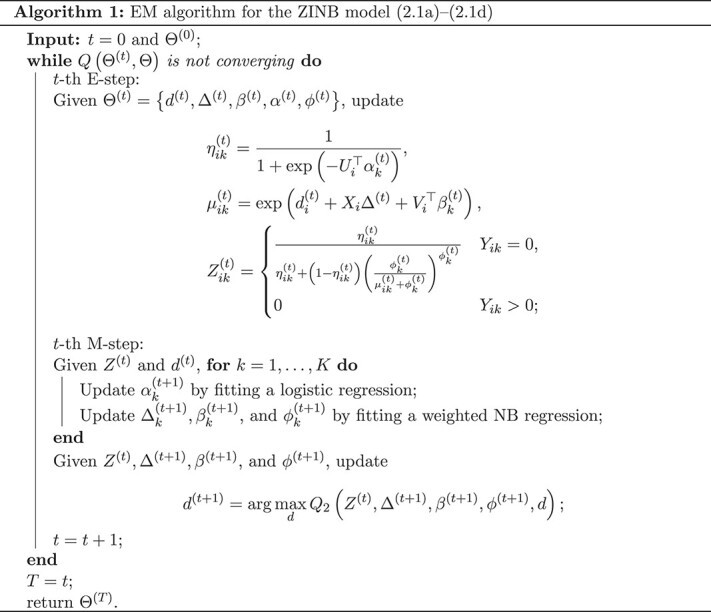


### Bias correction

**Figure 2:**
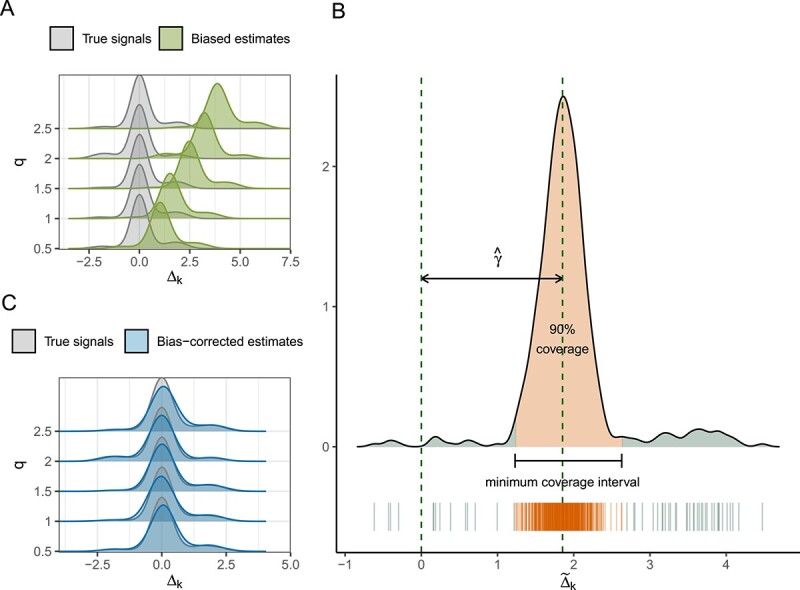
**Illustration of the bias correction step of mbDecoda.** (A) Visualization of the distribution of $\{\Delta _{1}, \ldots , \Delta _{K} \}$ (gray) and that of $\{\tilde{\Delta }_{1}, \ldots , \tilde{\Delta }_{K}\}$ (green) on five simulated examples with increasing level $q \in \{0.5,1,1.5,2,2.5\}$ of compositional bias. (B) Diagram of the MCI approach for bias correction. The majority of $\{\tilde{\Delta }_{1}, \ldots , \tilde{\Delta }_{K}\}$ (orange segments in the bottom scatter plot) tend to cluster together, leading to a spike in the empirical density curve. The MCI is defined to be the shortest continuous interval that covers a specific proportion (here 90% for illustration) of the area beneath the empirical density curve. Under the assumption of sparse signals, the estimates within MCI are expected to concentrate around zero, and the bias can thus be estimated using their mean value ($\hat{\gamma }$). (C) Visualization of the distribution of $\{\Delta _{1}, \ldots , \Delta _{K} \}$ (gray) and that of $\{\hat{\Delta }_{1}, \ldots , \hat{\Delta }_{K}\}$ (blue) on the same simulated examples as in (A). The gray and blue regions almost overlap, indicating that the MCI approach effectively rectified the bias.

Note that, for each $k$, $\log \left (\mu _{ik}\right )=\left (d_{i}-cX_{i}\right )+X_{i}\left (\Delta _{k}+c\right )+V_{i}^{\top }\beta _{k}$ holds for any $c$. Hence, the sample-specific parameter $d_{i}$ and the parameter of interest $\Delta _{k}$ are not identifiable. Consequently, the estimate of $\Delta _{k}$ obtained through Algorithm 1, $\tilde{\Delta }=\Delta ^{(T)}$, has an unknown bias $\gamma $. Figure 2(A) shows that the density curve of $\{\tilde{\Delta }_{1}, \ldots , \tilde{\Delta }_{K}\}$ and that of $\{\Delta _{1}, \ldots , \Delta _{K}\}$ exhibit a similar shape but with a noticeable shift, which can be attributed to the bias. To facilitate meaningful inference, bias correction is necessary.

In absolute abundance analysis, it is commonly assumed that only a small portion of taxa are truly associated with the variable of interest [15, 18, 19]. In other words, $\Delta $ is a sparse vector containing a large portion of zeroes. Under the sparsity assumption, the majority of $\tilde{\Delta }_{k}$ tend to cluster around $\gamma $. This inspires a novel method for estimating and correcting the bias. The idea is as follows. First, for a prespecified proportion $\rho $, we solve 


\begin{align*}& k^{*}=\mathop{\arg\min}\limits_{k:1\leq k \leq n-\lceil \rho k \rceil}\left(\tilde{\Delta}_{(k+\lceil \rho k \rceil)}-\tilde{\Delta}_{(k)}\right), \end{align*}

and construct the interval 


\begin{align*}& \left[\tilde{\Delta}_{(k^{*})},\tilde{\Delta}_{(k^{*}+\lceil \rho k \rceil)}\right], \end{align*}

where $\tilde{\Delta }_{(k)}$ denotes the $k$th smallest of $\tilde{\Delta }_{1}, \ldots , \tilde{\Delta }_{K}$ and $\lceil \rho k \rceil $ is the smallest integer that is greater than or equal to $\rho k$. For sufficiently large $K$, this amounts to approximately searching for the shortest continuous interval that covers a $100\times \rho \%$ of the area under the density curve of $\{\tilde{\Delta }_{1}, \ldots , \tilde{\Delta }_{K}\}$. Let $C_{0}$ denote the set of taxa that fall into this interval; we then estimate the bias by 


\begin{align*}& \hat{\gamma}=\frac{1}{\lceil \rho k \rceil}{\sum_{k\in C_{0}}\tilde{\Delta}_{k}}. \end{align*}

Finally, we obtain the de-biased estimate of $\Delta _{k}$ as 


\begin{align*}& \hat{\Delta}_{k}=\tilde{\Delta}_{k}-\hat{\gamma}. \end{align*}

We call this procedure the minimum coverage interval (MCI) approach, as illustrated in Figure 2(B). The effectiveness of MCI is demonstrated in Figure 2(C). Simulation results in the Supplementary Information illustrate that the performance of our method is not sensitive to the choice of $\rho $, when the signals are non-dense, and more so when most taxa are non-differentially abundant. In the absence of prior knowledge, we adopt $\rho =0.5$ as a default value.

### Inference

For each taxon $k$, we need to test the hypothesis 


\begin{align*}& H_{k 0}: \Delta_{k}=0 \quad versus \quad H_{k 1}: \Delta_{k}\neq 0. \end{align*}

To this end, we compute a t-statistic, 


\begin{align*} & t_k=\frac{\hat{\Delta}_k}{\text{SE}(\hat{\Delta}_k)}, \end{align*}

where $\mathrm{SE}(\hat{\Delta }_{k})$ denotes the standard error of $\hat{\Delta }_{k}$.

Note that 


\begin{align*}& \mathrm{var}( \hat{\Delta}_{k} )=\mathrm{var}( \tilde{\Delta}_{k} )+\mathrm{var}( \hat{\gamma} )-2\mathrm{cov}(\tilde{\Delta}_{k},\hat{\gamma}). \end{align*}

The first term $\mathrm{var}( \tilde{\Delta }_{k} )$ can be estimated through weighted NB regression when Algorithm 1 converges. We estimate the second term simply by 


\begin{align*}& \hat{\mathrm{var}}( \hat{\gamma} )=\frac{1}{\lceil \rho k \rceil}{\sum_{k\in C_{0}}(\tilde{\Delta}_{k}-\hat{\gamma})^{2}}. \end{align*}

Ignoring the last term leads to the modified t-statistic 


\begin{align*}& t_{k}=\frac{\hat{\Delta}_{k}}{\sqrt{\hat{\mathrm{var}}( \tilde{\Delta}_{k} )+\hat{\mathrm{var}}( \hat{\gamma} )}}. \end{align*}

To see why this might make sense, let us assume that $\hat{\Delta }_{1}^{*},\ldots , \hat{\Delta }_{K}^{*}$ are independent. Then 


\begin{align*}& \mathrm{cov}(\tilde{\Delta}_{k},\hat{\gamma})=\frac{1}{\lceil \rho k \rceil}\mathrm{var}( \tilde{\Delta}_{k} ). \end{align*}

For sufficiently large $\lceil \rho k \rceil $, the third term is negligible compared with the first term.

## RESULTS

### Simulation studies

We used simulated examples, with data generated from either the ZINB model or other models, to comprehensively evaluate the effectiveness and robustness of our proposed method, mbDecoda and compared it with ANCOMBC, fastANCOM, LinDA and LOCOM. ANCOM was excluded from the comparison, as it does not output P-values. Note that, in addition to Table 1, detailed software and version information for the competing methods can be found in the Supplementary Information. We adjusted P-values by the Benjamini–Hochberg (BH) procedure, and measured the performance of a method in terms of its power and empirical false discovery rate (FDR). All results were based on 100 replications.

#### Simulated data from the proposed ZINB model

For the sake of simplicity, we first considered the scenario where the variable of interest was binary and balanced, and there were no confounders. Denote by $n$ the sample size and $K$ the number of taxa. We set $\left (n,K\right ) \in \{\left (30,100\right ),\left (50,100\right ),\left (50,200\right )\}$ to examine three data configurations. The baseline absolute abundance, $\mathrm{exp}\left (\beta _{0k}\right )$, was drawn from a gamma mixture distribution, $0.6\cdot \mathrm{Gamma}\left (50,1\right )+0.3\cdot \mathrm{Gamma}\left (200,1\right )+0.1\cdot \mathrm{Gamma}\left (10\,000,1\right )$. Let $S$ denote the set of taxa that are truly associated with the variable of interest, and let $\pi $ denote the signal proportion. We varied the signal proportion, $\pi \in \{0.05,0.1,0.2,0.5\}$, and simply set $S=\{1,2,\ldots ,\pi K\}$. For each $k \notin S$, $\Delta _{k}=0$ by definition. For $k \in S$, $\mathrm{exp}\left (\Delta _{k}\right )$ was generated uniformly from the interval $\left (10^{-1},1.5^{-1}\right )$ or $\left (1.5,10\right )$. The sample-specific term $d_{i}$ was sampled from a normal distribution with mean $0.5\cdot X_{i}\left (\pi K\right )^{-1}\sum _{k\in S} |\Delta _{k}|$ and variance 1. Finally, we took the dispersion parameter $\phi _{k}=3$ and varied the zero-inflation parameter $\eta _{ik} \in \{0,0.1,0.3,0.5\}$.

**Figure 3:**
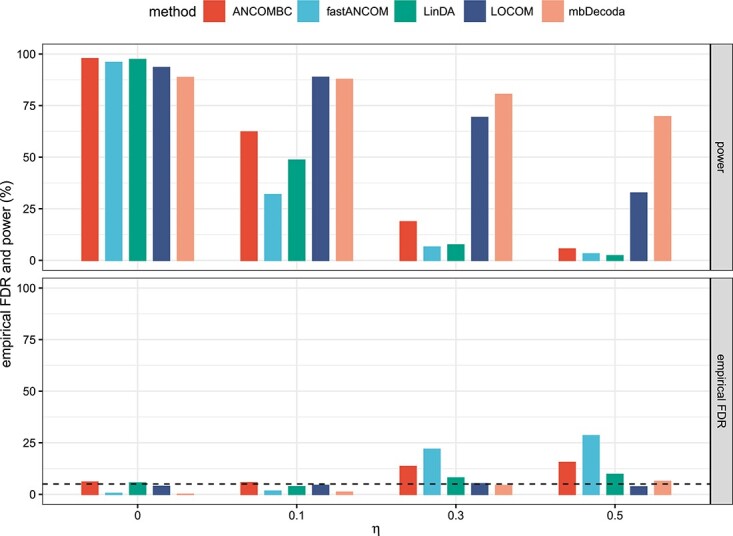
The average power and empirical FDR of various methods on data simulated from the ZINB model, in which the variable of interest was binary and there were no confounders, with $\left (n,K\right )=\left (50,100\right )$, $\pi =0.2$ and $\eta \in \{0,0.1,0.3,0.5\}$. The 5% nominal level of FDR is indicated by a dashed line.

Simulation results for varied levels of zero-inflation parameter, $\eta _{ik} \in \{0,0.1,0.3,0.5\}$, and a fixed signal proportion $\pi =0.2$ are shown in Figure 3 and Figures S1, S2, respectively, for $\left (n,K\right )=\left (50,100\right )$, $\left (n,K\right )=\left (30,100\right )$ and $\left (n,K\right )=\left (50,200\right )$. We can see that, when zero inflation was absent ($\eta _{ik} = 0$), all methods controlled the FDR very well while maintaining high power. However, when the degree of zero inflation was high, ANCOMBC, fastANCOM and LinDA exhibited alarmingly low power, and only mbDecoda and LOCOM effectively controlled the FDR at or near the specified nominal level. As the degree of zero-inflation increased, the power of mbDecoda and LOCOM decreased, with mbDecoda less affected than LOCOM. We can also see that the performance of each method improved as the sample size increased. Using the same simulated data, we also compared the performance of mbDecoda and three normalization-based methods, which address compositional bias by estimating scaling factors and incorporating them into a model-based approach. Further details can be found in the Supplementary Information. The results, depicted in Figure S3, highlight that our proposed method, mbDecoda, outperformed the normalization-based methods.

**Figure 4:**
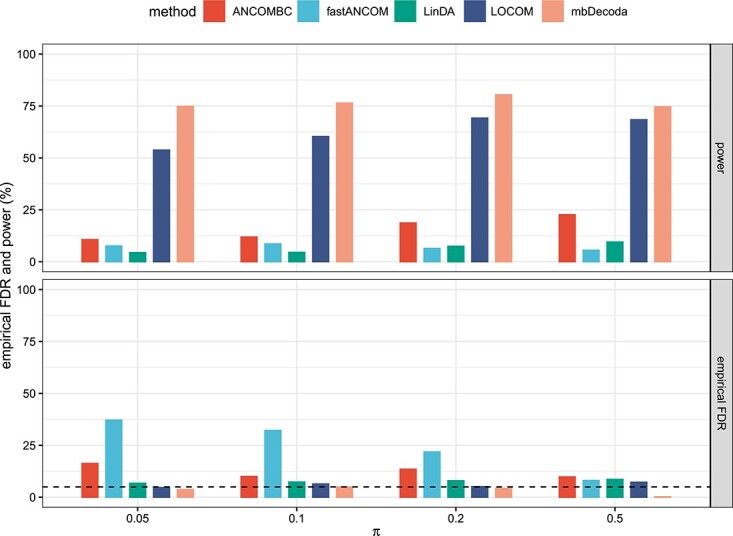
The average power and empirical FDR of various methods on data simulated from the ZINB model, in which the variable of interest was binary and there were no confounders, with $\left (n,K\right )=\left (50,100\right )$, $\eta =0.3$ and $\pi \in \{0.05,0.1,0.2,0.5\}$. The 5% nominal level of FDR is indicated by a dashed line.

Figure 4 and Figures S4, S5 show the results for varied signal proportion parameter, $\pi \in \{0.05,0.1,0.2,0.5\}$, with a fixed level of zero inflation $\eta _{ik}=0.3$, respectively, for $\left (n,K\right )=\left (50,100\right )$, $\left (n,K\right )=\left (30,100\right )$ and $\left (n,K\right )=\left (50,200\right )$. It is important to note that signal proportion and signal-to-noise ratio are distinct concepts, and the former is unrelated to the latter. We observe that ANCOMBC, fastANCOM and LinDA suffered a severe loss of power, mbDecoda had higher power than LOCOM and in most cases all methods, except for mbDecoda, failed to control the FDR. Furthermore, the power of each method increased with increasing sample size, and the performance of mbDecoda and LOCOM was more robust to the violation of the sparse signal assumption than other methods.

Additional simulation results, for $ (n,K ) \in \{ (50,20 ), (50,50 ), (100,50), (200,50 )\}$, are summarized in Table S1. It is notable that, with a relatively large sample size and small number of taxa, the conclusions remain qualitatively consistent, with mbDecoda demonstrating the best performance, followed by LOCOM. We further examined three simulation scenarios, keeping the same settings as in the previous scenario but with some modifications. In the first scenario, we enlarged the bias by drawing the sample-specific term $d_{i}$ from a normal distribution with mean $5\cdot X_{i}\left (\pi K\right )^{-1}\sum _{k\in S} |\Delta _{k}|$ and variance 1. In the second scenario, the variable of interest had a standard normal distribution. In the third scenario, there were two confounders in the negative binomial part. One followed a uniform distribution within the interval $\left (0,1\right )$, while the other had a Bernoulli distribution with a parameter of 0.5. These confounding factors had coefficients of 1 and -1, respectively. For simplicity, we set $\left (n,K\right )=\left (50,100\right )$, $\pi =0.2$ and $\eta \in \{0,0.1,0.3,0.5\}$. The results are shown in Figures S6–S8. When combined with Figure 3, it is evident that the performance of mbDecoda remained largely unaffected by the magnitude of compositional bias (that is, the mean of the sample-specific term $d_{i}$), while this bias had an adverse effect on the power of LOCOM and on the empirical FDR of ANCOMBC, fastANCOM and LinDA. Furthermore, the presence of confounding factors led to a substantial loss of power for LOCOM, while it had very limited effect on the performance of other methods. Finally, when the type of the variable of interest was continuous, LOCOM suffered a loss of power, and failed to control the FDR, while the performance of mbDecoda remained the best.

So far, in the simulation, a fixed zero-inflation rate was assigned to all taxa, which is unrealistic. Although ZINB is appropriate for some taxa, there is evidence showing that NB is for others [29]. To provide a more fair and objective assessment of mbDecoda and its competitors, we conducted an additional experiment with mixed levels of zero-inflation. Specifically, 50% of the taxa display no zero inflation, while the remaining 50% exhibit zero-inflation rates uniformly distributed between 0 and 0.7. The simulation results are summarized in Table S2. It is evident that mbDecoda exhibited the best performance, demonstrating its effectiveness in handling mixed levels of zero-inflation.

#### Simulated data from other models

To ensure a fair comparison with other methods, we also explored alternative data generation processes as follows.

(M1) An alternative model, denoted as ZIP, that replaces the negative binomial distribution in the ZINB model with a Poisson distribution.

(M2) One simulated example employed by ANCOMBC, where the absolute abundance data were drawn from a Poisson–Gamma mixture distribution, and the observed count table from a multinomial distribution.

(M3) The data generation process used by LinDA, where the absolute abundance data were generated from a log-normal distribution, and then the observed count matrix from a multinomial distribution.

(M4) The simulation framework described in Mcmurdie & Holmes [30]. It sampled abundance data from a multinomial distribution and multiplied randomly selected microbes by an effect size. The multinomial parameters were learned from real microbiome data.

We set $\left (n,K\right )=\left (30,100\right )$ for models M1–M4, and took the signal proportion $\pi $ to be 0.2. See the Supplementary Information for details of the simulation settings.

**Figure 5:**
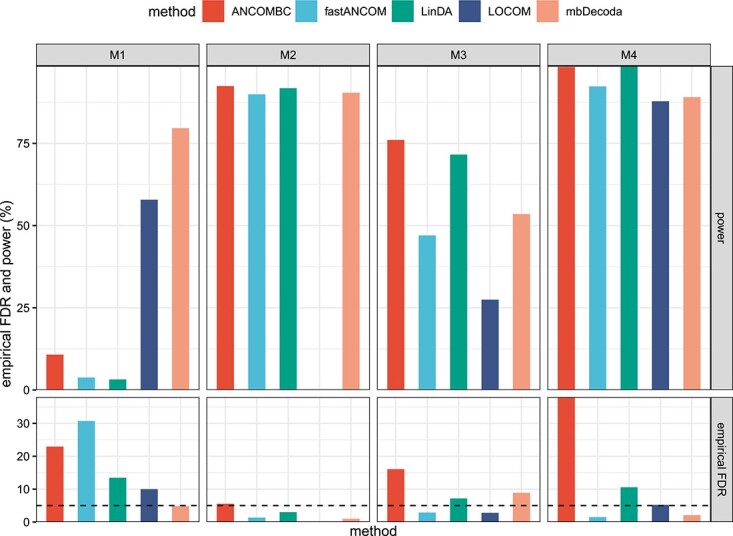
The average power and empirical FDR of various methods on data simulated from other models, M1–M4. The 5% nominal level of FDR is indicated by a dashed line.

The results are shown in Figure 5. When data were generated from the ZIP model (M1), mbDecoda maintained considerably high power compared with ANCOMBC, fastANCOM and LinDA, while all other methods failed to control the FDR. As expected, ANCOMBC performed well on its own simulation setting (M2). fastANCOM, LinDA and mbDecoda performed comparatively well. Unfortunately, LOCOM reported errors on this scenario, possibly due to the presence of 20% structural zeros. LinDA performed better than other methods on its own simulation setting (M3), followed by fastANCOM and mbDecoda. ANCOMBC exhibited a higher empirical FDR, and LOCOM suffered a loss of power. Finally, in simulation setting (M4), fastANCOM, LOCOM and mbDecoda controlled the FDR well while maintaining high power, and ANCOMBC had highly inflated empirical FDR.

In summary, the performance of mbDecoda was highly robust to the model misspecification. In the presence of zero-inflation (M1), ANCOMBC, fastANCOM and LinDA suffered empirical FDR inflation, and had lower power than LOCOM and mbDecoda. This is consistent with the conclusions drawn from simulations under the ZINB model, suggesting the necessity of accounting for zero-inflation. As in the ZINB simulation setting, LOCOM performed poorly when the sample size was small.

### Real data applications

We utilized real datasets to assess the performance of mbDecoda and its competitors across various metrics. Specifically, we evaluated their effectiveness in terms of type I error control using the Human Microbiome Project (HMP) healthy cohort dataset, examined their reproducibility across multiple datasets for the same disease and assessed their ability to detect differentially abundant taxa between obesity or type 2 diabetes and control groups.

#### Assessment of type I error control

Since parametric models may fail to capture the full complexity of microbiome sequencing counts, it is important to study type I error control on real data. Therefore, we conducted an experiment to assess our method and its competitors using data from the HMP healthy cohort [31]. We concentrated on the class level and excluded taxa present in less than 5% of samples, resulting in a dataset with 748 samples and 18 taxa. Note that filtering rare taxa is a common preprocessing step in microbiome data analysis aimed at reducing noise. In principle, this preprocessing step is unnecessary for the proposed method. However, for competing methods such as ANCOM and fastANCOM, which are unable to handle zeros, filtering taxa with a prevalence of less than 5% ensures a more fair comparison among all methods.

Since the ground truth is unknown for real data, we shuffled the sample labels to create a global null situation. For each taxon, the process was repeated 100 times, so that the assessment of type I error is possible. At a nominal significance level of 0.05, the results are shown in Figures S10, S11. We can see that fastANCOM was overly conservative, while LinDA and ANCOMBC, without FDR control, had inflated type I error. In contrast, both mbDecoda and LOCOM demonstrated better control over the type I error at the nominal level.

#### Consistency assessment

Conclusions drawn from microbiome studies often exhibit a lack of consistency, which could stem from various factors such as intricate nature of microbiome data, biases introduced during the sequencing process and the combined effects of sample size and selection. Here, we carried out absolute abundance analysis using publicly available data from published studies to evaluate the reproducibility of our method and its competitors. Six datasets were acquired from either the MicrobiomeHD [32] or the curatedMetagenomicData [33] database, where raw sequencing data for each study were downloaded and processed through a standardized pipeline. The basic characteristics for these datasets are summarized in Table 2. We consolidated taxa at genus level and filtered taxa with prevalence less than 5%. The variable of interest is binary indicating either the diseased or healthy status. We applied mbDecoda and other methods to identify differentially abundant taxa between samples from these two groups, and controlled the FDR at level 0.05 by the BH procedure.

**Table 2:** Summary of microbiome datasets used in consistency assessment. The last two columns denote the number of genera ($K$) and the total number of samples ($n$). The variable of interest is binary, indicating the control status, and the goal is to compare abundance levels, taxon-by taxon, between the two groups

Case	Disease	Reference	Database	$\boldsymbol K$	$\boldsymbol n$
A	CRC	Baxter et al. [34]	MicrobiomeHD	139	292
		Zackular et al. [35]	MicrobiomeHD	139	60
	CDI	Schubert et al. [36]	MicrobiomeHD	92	247
		Vincent et al. [37]	MicrobiomeHD	92	50
B	IGT	Karlsson et al. [38]	curatedMetagenomicData	104	92 (full set)
				104	46 (subset)
	ME/CFS	Nagy-Szakal et al. [39]	curatedMetagenomicData	89	100 (full set)
				89	50 (subset)

Case A: studies on colorectal cancer (CRC) and Clostridium difficile infection (CDI). For each disease, two datasets, distinguished by disparate sample sizes—one large and one small—were obtained from the MicrobiomeHD database. We assessed the consistency of the detected taxa on the two datasets by calculating the Jaccard index, which quantifies the similarity between two sets by calculating the ratio of the size of their intersection to the size of their union.

Case B: studies on impaired glucose tolerance (IGT) and myalgic encephalomyelitis/chronic fatigue syndrome (ME/CFS). For each disease, we acquired the dataset from the curatedMetagenomicData database. For each dataset, we randomly chose half of the samples to construct a data subset. We then assessed the consistency of the detected taxa on the full set and the subset.

**Figure 6:**
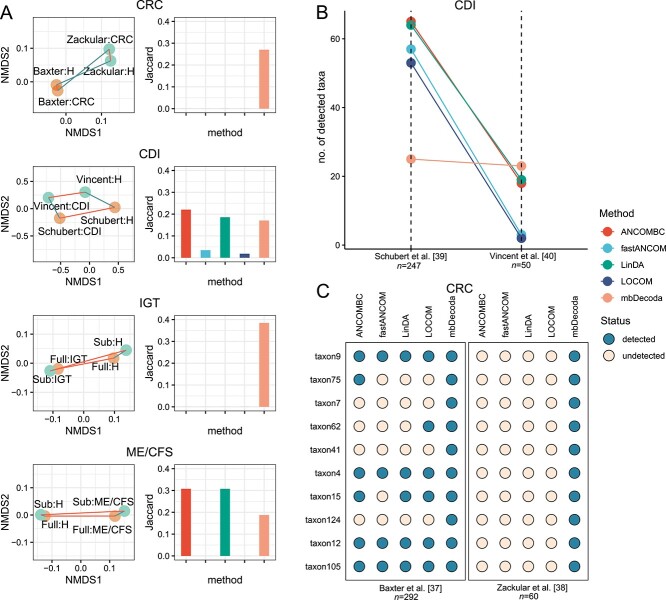
**Consistency assessment of various absolute abundance analysis methods across different datasets.** (A) Two-dimensional non-metric multidimensional scaling (NMDS) plots of the microbiomes, with centroids indicated by data subsets, and bar plots of the Jaccard index for measuring the consistency of the detected taxa on two datasets, with a significance level of 0.05. (B) Plot of the number of taxa detected by each method on two CDI datasets with different sample sizes. (C) Visualization of the 10 taxa consistently detected by mbDecoda across two CRC datasets, along with their detection status by other methods.

The left panels of Figure 6(A) demonstrate that, for both diseases IGT and ME/CFS, the subset and the full set were intentionally designed to have similar distributions. However, for the disease CRC, a notable level of inter-study heterogeneity is observed, characterized by a substantial gap between the two studies compared with the distance between the diseased and healthy groups within each study. A similar pattern can be seen for the disease CDI, although it is less pronounced. The results for the Jaccard index are shown in the right panels of Figure 6(A). We see that, for all the datasets, mbDecoda had a certain level of reproducibility. However, for the CRC and IGT datasets, all other methods were not able to reproduce any results, and for the CDI and ME/CFS datasets, mbDecoda, ANCOMBC and LinDA behaved similarly and were significantly better than fastANCOM and LOCOM. Figure 6(B) demonstrates that, in terms of the number of detected taxa, fastANCOM and LOCOM were very conservative when the sample size was small, and that ANCOMBC and LinDA were very aggressive when the sample size was large. In contrast, the performance of mbDecoda was robust to a decrease in the sample size. Figure 6(C) shows that a total of 10 differentially abundant taxa were identified by mbDecoda in both the small and large CRC datasets, and most of them were detected by at least one of the other methods in the large dataset. However, none of these taxa were identified as differentially abundant by all other methods when the sample size was small. Overall, in terms of consistency assessment, mbDecoda performed the best.

#### Exploring gut microbiota in metabolic disorders

Recent research has indicated that the gut microbiota plays a crucial role in host energy metabolism, immune function regulation and metabolic disorders [40]. Previous studies have observed differences in the gut microbiota composition between obese (OB) and type 2 diabetes (T2D) patients compared with healthy individuals [41, 42].

We acquired one OB dataset [43] and one T2D dataset [44], respectively, from the MicrobiomeHD and curatedMetagenomicData databases. We focused on the genus level and excluded taxa that appeared in less than 5% of the samples. The basic characteristics for the two datasets are summarized in Table 3. The OB dataset was from a microbiome study of the Amish population with strong lifestyle homogeneity, and the T2D dataset involved fecal samples of Chinese individuals diagnosed with T2D and their non-diabetic controls. We applied mbDecoda and other methods to detect differentially abundant taxa between the healthy and diseased populations. Gender in the OB dataset and gut enterotype in the T2D dataset were each treated as a confounding variable and then adjusted accordingly. We controlled the FDR at level 0.05 by the BH procedure.

**Table 3:** Characteristics of the gut microbiota datasets for obesity and type 2 diabetes. The last four columns denote the number of genera ($K$), the total number of samples ($n$), the variable of interest ($X$) and the confounding variable ($V$). The goal is to compare abundance levels, taxon-by-taxon, between the diseased and control groups

Disease	Reference	Database	$\boldsymbol K$	$\boldsymbol n$	$\boldsymbol X$	$\boldsymbol V$
OB	Zupancic *et al.* [ [43]	MicrobiomeHD	97	169	OB/control	gender
T2D	Qin *et al.* [44]	curatedMetagenomicData	111	343	T2D/control	gut enterotype

**Figure 7:**
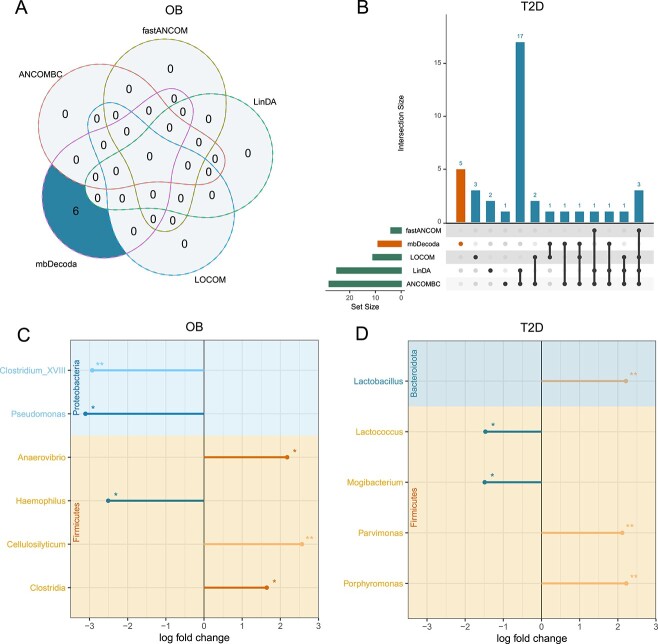
**Differential abundance analysis of the OB and T2D datasets.** (**A**) Venn diagram of differentially abundant taxa between OB and control groups with a nominal FDR level of 0.05. (**B**) Visualization of detected taxa between T2D and control groups with a nominal FDR level of 0.05. (**C**) Plot of the log fold change for differentially abundant taxa between OB and control groups detected by mbDecoda. (**D**) Plot of the log fold change for differentially abundant taxa between T2D and control groups uniquely detected by mbDecoda. *adjusted P-values were between 0.01 and 0.05, **adjusted P-values were less than 0.01.

For the OB dataset, as illustrated in Figure 7(A), mbDecoda identified six taxa that were significantly associated with obesity, but none of the other methods were able to identify any significant associations. These six taxa are displayed in Figure 7(C). Among these, *Anaerovibrio* and *Clostridia* have been previously linked to obesity [45–47], and *Pseudomonas* and *Haemophilus* have been implicated in dietary energy intake, suggesting their potential role in influencing obesity [48, 49].

For the T2D dataset, as shown in Figure 7(B), ANCOMBC and LinDA detected a notably larger number of taxa compared with the other methods. This aggressive behavior on this large dataset was also observed in the consistency assessment section. Considering the inflation of FDR for ANCOMBC and LinDA, as indicated by simulation studies, it is plausible that many of these identified taxa were likely false discoveries, despite of a substantial overlap in the taxa detected by both methods. In contrast, fastANCOM was very conservative, which was consistent with its performance in simulation studies. The behavior of mbDecoda and LOCOM was intermediate compared with the other methods, and as shown in Figure 7(D), mbDecoda identified five unique taxa. Among these, *Mogibacterium* has been reported as an opportunistic pathogen in T2D [50], and *Lactobacillus* and *Porphyromonas* have been suggested to have associations with T2D [51–53]. Furthermore, *Lactococcus* has been linked to a high-fat diet, indicating its strong association with obese and diabetic phenotypes [54, 55].

## DISCUSSION

High-throughput sequencing technologies and modern bioinformatics tools have been fundamental in understanding the structure and function of healthy microbial communities, as well as unraveling the associations between microbiota and various conditions and diseases. Potentially pathogenic or probiotic microbes can be identified by comparing their abundance levels between healthy and diseased populations, or more broadly, by linking microbiome composition with clinical phenotypes or environmental factors. While the analysis of microbiome compositions is crucial in this context, it is important to note that the compositionality, sparsity and over-dispersion of microbiome abundance data can introduce biases or yield misleading results when classical data analysis methods are employed.

To overcome these challenges, we have introduced a novel model-based method called mbDecoda. mbDecoda describes sparse, over-dispersed and count-valued abundance data using a ZINB model. The structural form of the model captures how the mean abundance changes according to levels of the variable of interest while controlling effects of possible confounding variables. Our methodology includes the development of an efficient EM algorithm for estimating model parameters, a bias correction strategy based on the concept of an MCI and a modified t-statistic for conducting association analysis at the absolute abundance level.

Extensive simulation studies were carried out to assess the performance of mbDecoda and other methods for absolute abundance analysis. The findings show that mbDecoda was the best performer, and its performance was robust to model misspecification or a decrease in the sample size. Additionally, these methods were applied to real-world microbiome datasets, with emphasis on evaluating their consistency across different datasets. mbDecoda demonstrated a certain level of reproducibility. In summary, these results indicate mbDecoda as a reliable method for absolute abundance analysis.

The proposed method still has certain limitations that warrant attention. Firstly, apart from addressing issues such as compositionality, sparsity and over-dispersion, it is crucial to consider the intricate correlation structure among taxa. Therefore, there is a need to extend the univariate and taxon-by-taxon approach to simultaneously handle multiple taxa, thus leveraging the interdependence among microbes. Secondly, while cross-sectional studies are valuable for identifying differences in microbial communities between different conditions, the substantial variation in the microbiome among individuals necessitates the use of longitudinal studies in microbiome research. However, observations from repeated measures in longitudinal studies exhibit correlation, and an important avenue for future research is to extend mbDecoda from analyzing cross-sectional data to longitudinal data, thereby accommodating correlated observations over time.

**Key Points**

- We reviewed statistical methods for testing differential abundance in microbiome studies. These methods utilize observed relative abundances to make inferences about unobservable absolute abundances.
- We introduced mbDecoda, a model-based framework for analyzing compositions of microbiomes. mbDecoda employs the ZINB model to address issues related to compositionality, sparsity and overdispersion, eliminating the need for data normalization and transformation.
- The key components of mbDecoda include an EM algorithm for parameter estimation, an MCI strategy for correcting compositional bias and a modified t-test for absolute abundance analysis.
- We demonstrated that mbDecoda is an accurate and reliable method for absolute abundance analysis. It is expected to be a valuable addition to the statistical toolbox for analyzing and interpreting microbiome data.

## Supplementary Material

supp_mbDecoda_bbae205

## Data Availability

We downloaded the CRC and CDI datasets from https://zenodo.org/record/569601 and used the function *curatedMetagenomicData* from the R package **curatedMetagenomicData** (version 3.6.2) to get the IGT and ME/CFS datasets. The source code and data for reproducing main results in the paper are available at https://github.com/YuxuanZ0ng/mbDecoda.

## Author Biographies

**Yuxuan Zong** is a PhD candidate in the SJTU-Yale Joint Center of Biostatistics and Data Science at Shanghai Jiao Tong University. Her research interests lie in statistical modeling and analysis of microbiome data.

**Hongyu Zhao** is Ira V. Hiscock professor of Biostatistics, professor of Genetics and of Statistics and Data Science at Yale University. His research interests include statistical genomics and proteomics, microbiome, data integration, high dimensional data, and network and graphical models.

**Tao Wang** is a professor in the SJTU-Yale Joint Center of Biostatistics and Data Science at Shanghai Jiao Tong University. His research interests include statistics for high-dimensional data, biostatistics, and statistical machine learning.
