# Supplementary material for: mbDecoda: a debiased approach to compositional data analysis for microbiome surveys
Source: Brief Bioinform. 2024 May 2;25(3):bbae205. doi: 10.1093/bib/bbae205 (PMC11066923; doi:10.1093/bib/bbae205)
Supplement: supp_mbDecoda_bbae205 [file supp_mbdecoda_bbae205.pdf]

Supplementary Information

Software and version details

In this paper, all analyses were conducted using R version 4.2.2. Below, we describe some of the software and tools employed in our research.

ANCOMBC: We used the function *ancombc* from the R package **ANCOMBC** (version 1.2.2), and set p_adj_method = "BH", zero_cut = 1.1, and lib_cut = 1. No filtering of taxa or samples was applied.

LinDA: We used the function *linda* from the R package **MicrobiomeStat** (version 1.1) with default parameter settings. No filtering of taxa or samples was applied.

fastANCOM: We used the function *fastANCOM* from the R package **fastANCOM** (version 0.0.4), with a zero_cut parameter set to 1. No filtering of taxa or samples was applied.

LOCOM: We used the function *locom* from the R package **LOCOM** (version 1.1), and with parameters set to fdr.nominal = 0.05, prev.cut = 0, and seed = 1. No filtering of taxa or samples was applied.

Wrench: We used the function *wrench* from the R package **Wrench** (version 1.1.0) with default settings.

DESeq2: We used the function *DESeq* from the R package **DESeq2** (version 1.34.0), and set pAdjustMethod = "BH".

edgeR: We used the function *glmQLFTest* from the R package **edgeR** (version 3.36.0) with default settings.

The R file for the omnibus test was downloaded from `https://github.com/jchen1981/MicrobiomeDDA/blob/master/zeroinfl.plus.daa.R`. We used the function *ZISeq* and set size.factor = "NULL" (GMPR), winsor = "F", method = "omnibus", and filter = "F".

Simulated data from the proposed ZINB model: additional examples

Comparison with normalization-based methods: In the context of differential abundance testing, ZINB-based methods, such as the omnibus test by Chen *et al.* [56], typically address compositional bias by estimating scaling factors and incorporating them into the ZINB model. Various normalization methods exist for estimating scaling factors, including the geometric mean of pairwise ratios (GMPR) utilized by Chen *et al.* [6], the Wrench method proposed by Kumar *et al.* [5], and other methods referenced therein. Similar to the omnibus

test, Wrench can be paired with a model-based approach for conducting differential abundance analysis. To compare the performance of different methods, we conducted a simulation study involving mbDecoda, the omnibus test, Wrench+DESeq2, and Wrench+edgeR. It is important to note that both mbDecoda and the omnibus test utilize the ZINB distribution, whereas Wrench+DESeq2 and Wrench+edgeR are based on the NB distribution. The simulation setting was the same as that of **Figure 3** in the main text. The results of the simulation study, depicted in **Figure S3**, highlight that our proposed method, mbDecoda, outperformed the omnibus test, Wrench+DESeq2, and Wrench+edgeR.

Simulations with a relatively large sample size or a small number of taxa: Differential abundance analysis can be performed at high taxonomic levels, where the number of taxa may be small, and under a large sample size. We included four additional scenarios, maintaining the same settings as **Figure 3** in the main text, except that $(n, K) \in \{(50, 20), (50, 50), (100, 50), (200, 50)\}$ and $\eta = 0.3$. The results are summarized in **Table S1**. Notably, with a relatively large sample size and a small number of taxa, the conclusions remain qualitatively consistent, with mbDecoda demonstrating the best performance, followed by LOCOM.

Simulations with mixed levels of zero-inflation: Although ZINB is appropriate for some taxa, there is evidence showing that NB is suitable for others [29]. To provide a more fair and objective assessment of mbDecoda and its competitors, we conducted an additional experiment with mixed levels of zero-inflation. The simulation setting was the same as that of **Figure 3** in the main text, except that 50% of the taxa display no zero inflation, while the remaining 50% exhibit zero-inflation rates uniformly distributed between 0 and 0.7. The simulation results are summarized in **Table S2**. It is evident that mbDecoda exhibited the best performance, demonstrating its effectiveness in handling mixed levels of zero-inflation.

Choice of ρ for the MCI approach: Differential abundance testing typically assumes that signals are non-dense or even sparse, meaning that most taxa are non-differentially abundant. Using the same simulated data as that of **Figure 4** in the main text, **Figure S9** illustrates that the performance of our method is not sensitive to the choice of ρ, when the signals are non-dense, and more so when most taxa are non-differentially abundant. In the absence of prior knowledge, we adopt $\rho = 0.5$ as a default value.

Simulated data from other models: setting details

Simulation settings of the ZIP model (M1) The ZIP model replaces the negative binomial distribution of ZINB with the Poisson distribution. We considered the scenario where the variable of interest was binary, and there were no confounders. We set $\eta = 0.3, \pi = 0.2, (n, K) = (30, 100)$, with all other parameters being the same as in the ZINB setting.

Simulation settings of the data generation mechanism (M2) from Lin & Peddada [18] We downloaded the simulation code from https://github.com/FrederickHuangLin/ANCOM-BC,We explored the scenario with balanced library sizes, unbalanced microbial loads, and a large sampling fraction. Structural and outlier zeros were generated at proportions of 20% and 5%, respectively. We set the signal proportion to be 0.2, and $(n, K) = (30, 100)$.

Simulation settings of the data generation mechanism (M3) from Zhou *et al.* [19] We downloaded the simulation code from https://github.com/zhouhj1994/LinDA-manuscript-result. We explored the basic setting of LinDA without zero-inflation, referred to as C0S4, and set $\sigma^2 = 1$ and $\mu = 2$. We set the signal proportion to be 0.2, and $(n, K) = (30, 100)$.

Simulation settings of the data generation mechanism (M4) from Mcmurdie & Holmes [30] The simulation code was downloaded from https://doi.org/10.1371/journal.pcbi.1003531.s001. We set the biological source template as feces, with an effect size of 5. The total reads per sample was set as 10000. We set the signal proportion to be 0.2, and $(n, K) = (30, 100)$.

Supplemental figures

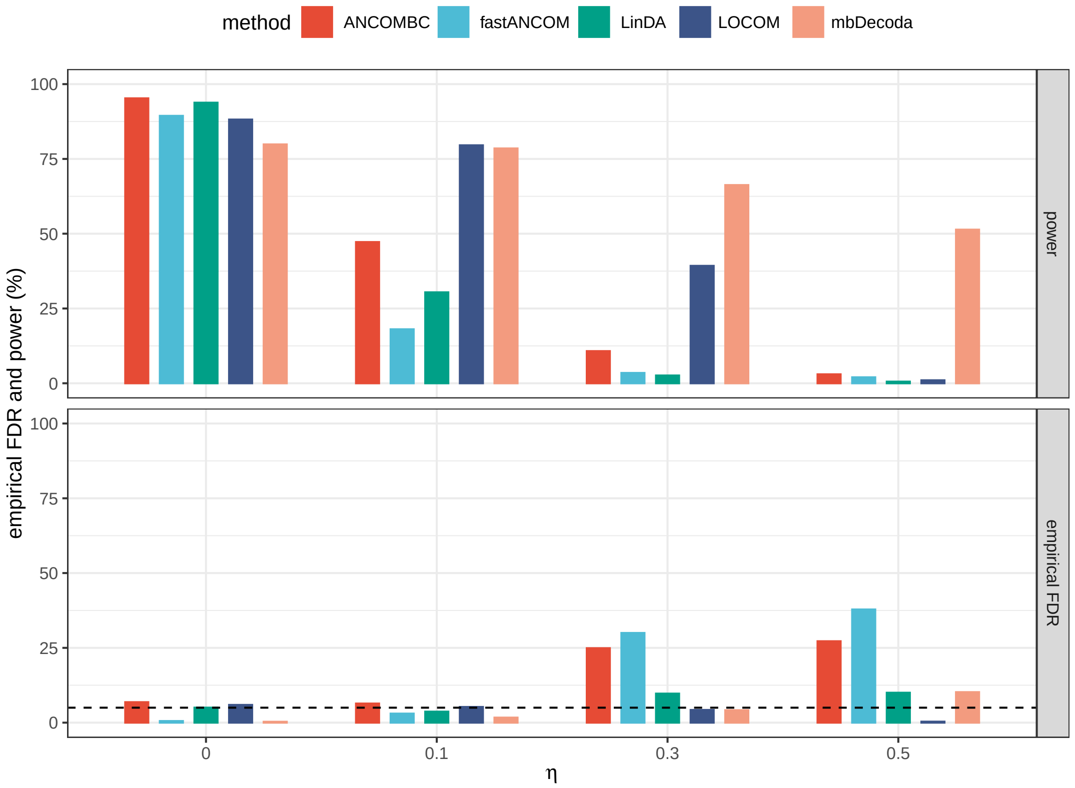

Figure S1: The average power and empirical FDR of various methods on data simulated from the ZINB model, in which the variable of interest was binary and there were no confounders, with $(n, K) = (30, 100)$, $\pi = 0.2$, and $\eta \in \{0, 0.1, 0.3, 0.5\}$. The 5% nominal level of FDR is indicated by a dashed line.

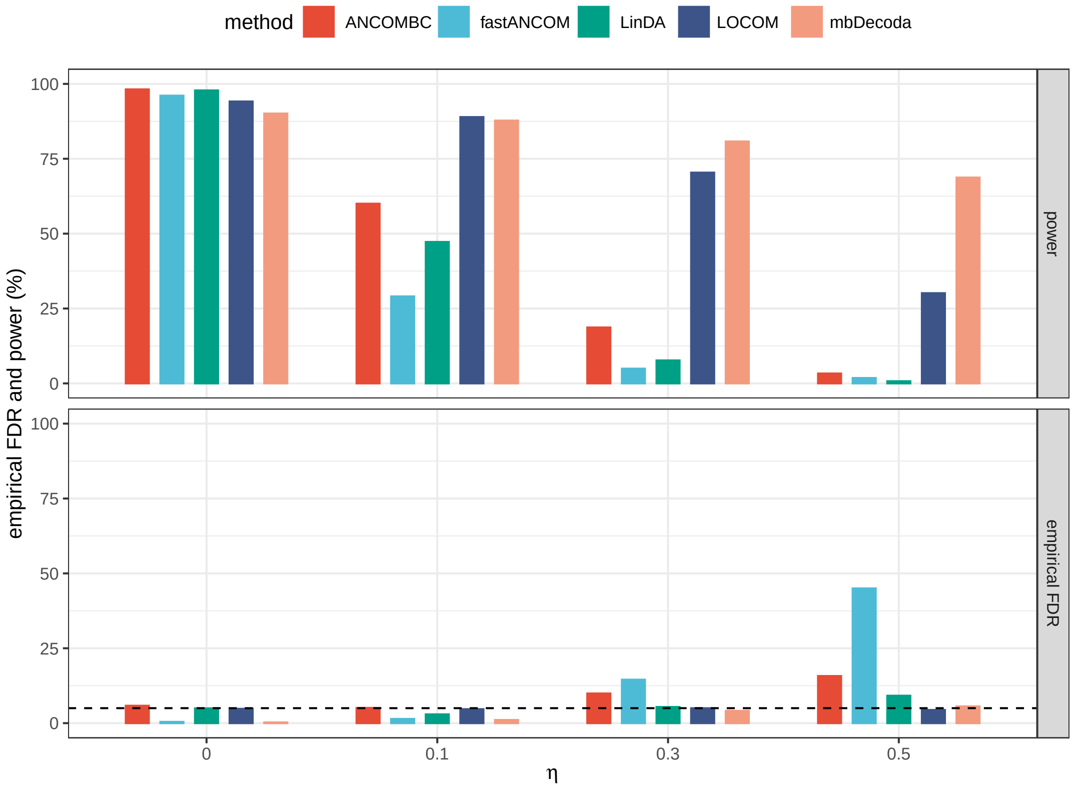

Figure S2: The average power and empirical FDR of various methods on data simulated from the ZINB model, in which the variable of interest was binary and there were no confounders, with $(n, K) = (50, 200)$, $\pi = 0.2$, and $\eta \in \{0, 0.1, 0.3, 0.5\}$. The 5% nominal level of FDR is indicated by a dashed line.

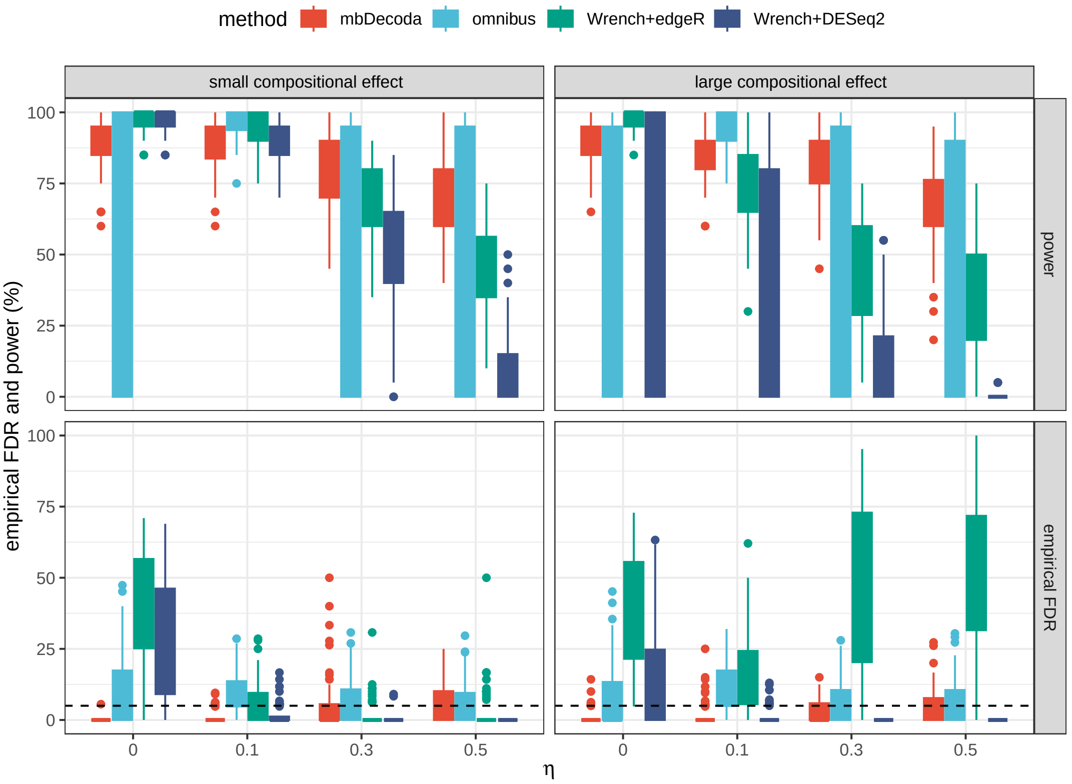

Figure S3: The average power and empirical FDR of various methods on data simulated from the ZINB model, in which the variable of interest was binary and there were no confounders, with $(n, K) = (50, 100)$, $\pi = 0.2$, and $\eta \in \{0, 0.1, 0.3, 0.5\}$. The 5% nominal level of FDR is indicated by a dashed line.

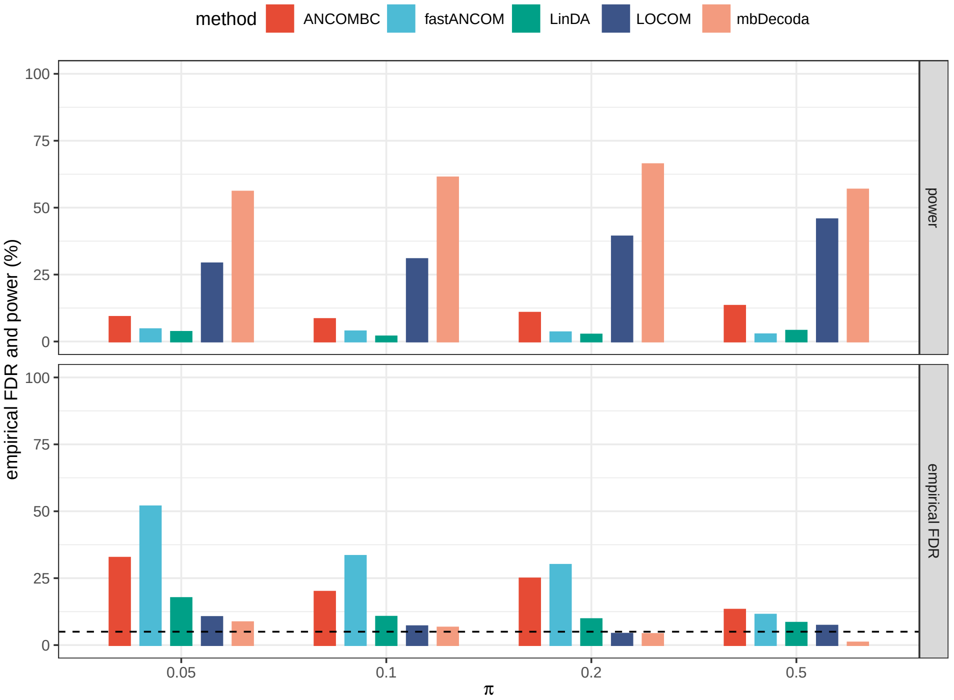

Figure S4: The average power and empirical FDR of various methods on data simulated from the ZINB model, in which the variable of interest was binary and there were no confounders, with $(n, K) = (30, 100)$, $\eta = 0.3$, and $\pi \in \{0.05, 0.1, 0.2, 0.5\}$. The 5% nominal level of FDR is indicated by a dashed line.

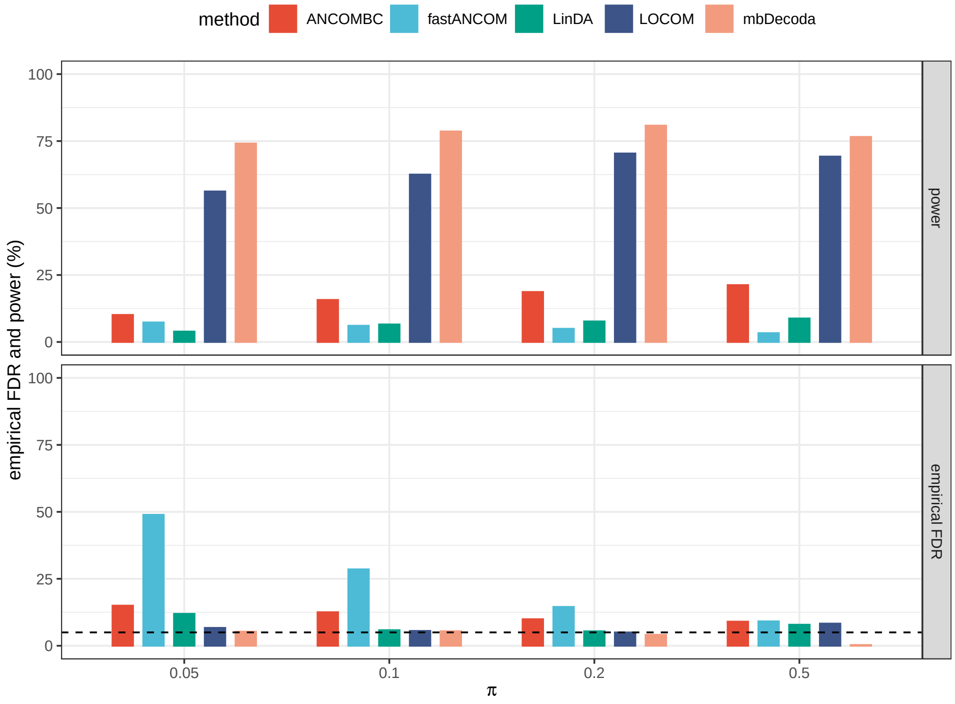

Figure S5: The average power and empirical FDR of various methods on data simulated from the ZINB model, in which the variable of interest was binary and there were no confounders, with $(n, K) = (50, 200)$, $\eta = 0.3$, and $\pi \in \{0.05, 0.1, 0.2, 0.5\}$. The 5% nominal level of FDR is indicated by a dashed line.

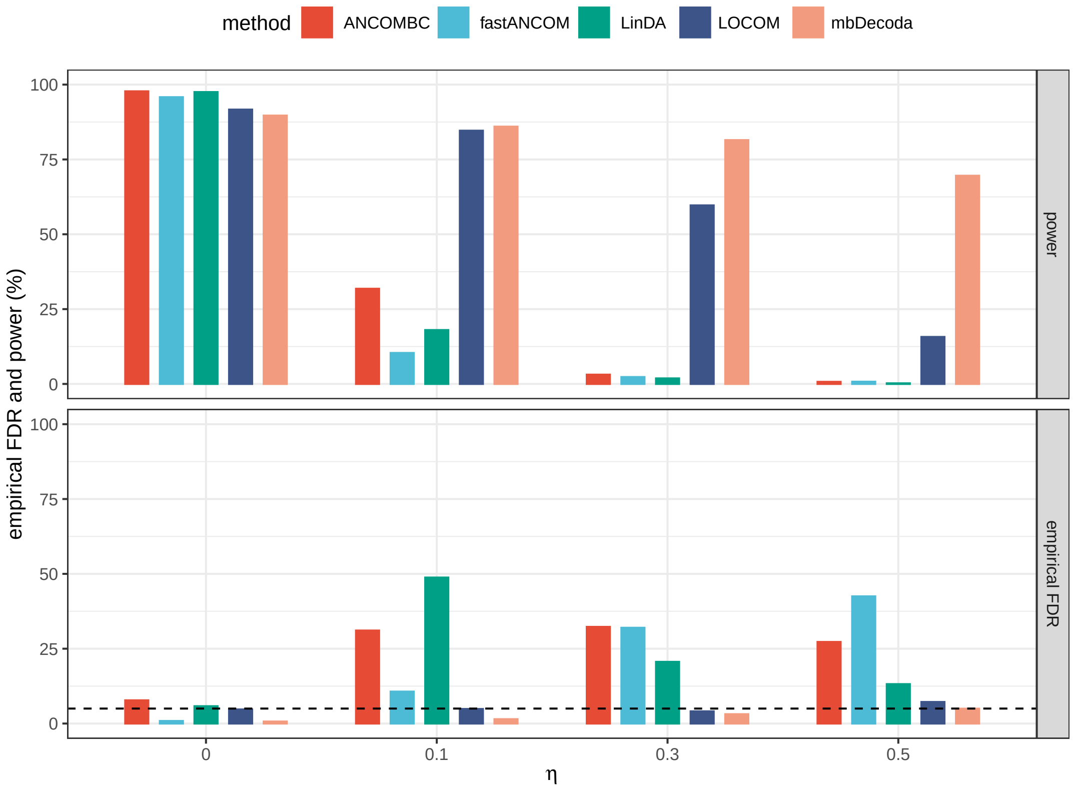

Figure S6: The average power and empirical FDR of various methods on data simulated from the ZINB model, in which the variable of interest was binary and there were no confounders, with $(n, K) = (50, 100)$, $\pi = 0.2$, $\eta \in \{0, 0.1, 0.3, 0.5\}$, and a large compositional effect. The 5% nominal level of FDR is indicated by a dashed line.

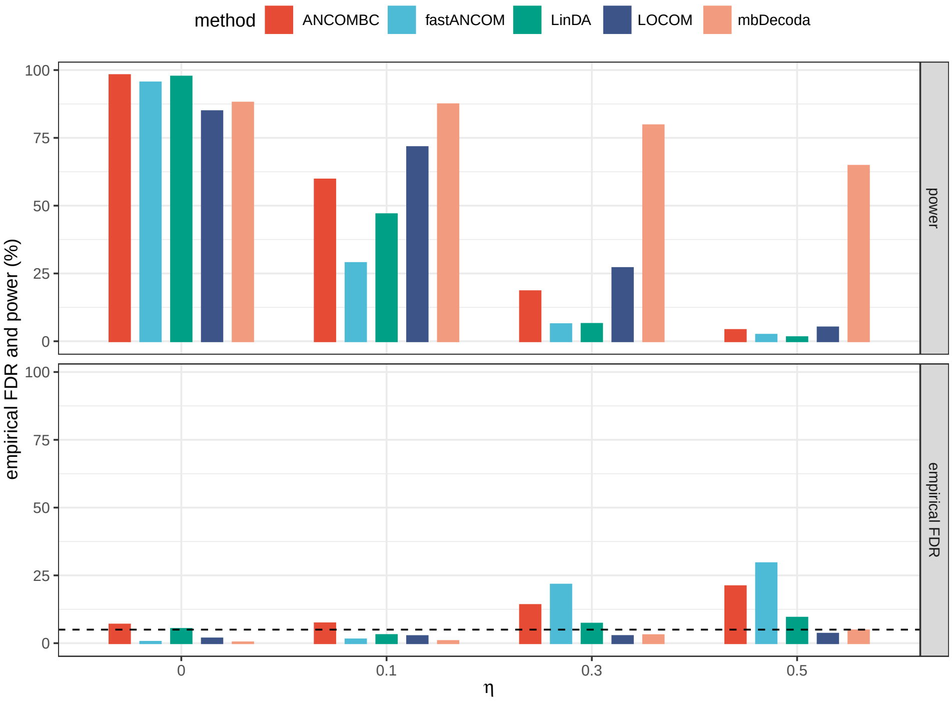

Figure S7: The average power and empirical FDR of various methods on data simulated from the ZINB model, in which the variable of interest was binary and there were two confounders, with $(n, K) = (50, 100)$, $\pi = 0.2$, and $\eta \in \{0, 0.1, 0.3, 0.5\}$. The 5% nominal level of FDR is indicated by a dashed line.

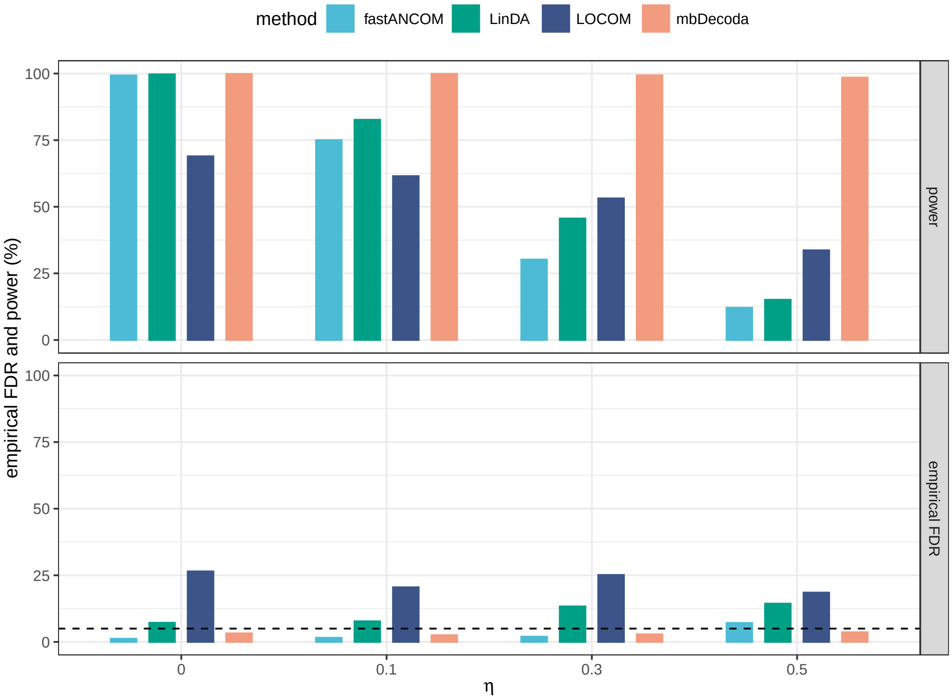

Figure S8: The average power and empirical FDR of various methods on data simulated from the ZINB model, in which the variable of interest was continuous and there were no confounders, with $(n, K) = (50, 100)$, $\pi = 0.2$, and $\eta \in \{0, 0.1, 0.3, 0.5\}$. The 5% nominal level of FDR is indicated by a dashed line. AMCOMBC was not included in the comparison as it can only handle discrete variables.

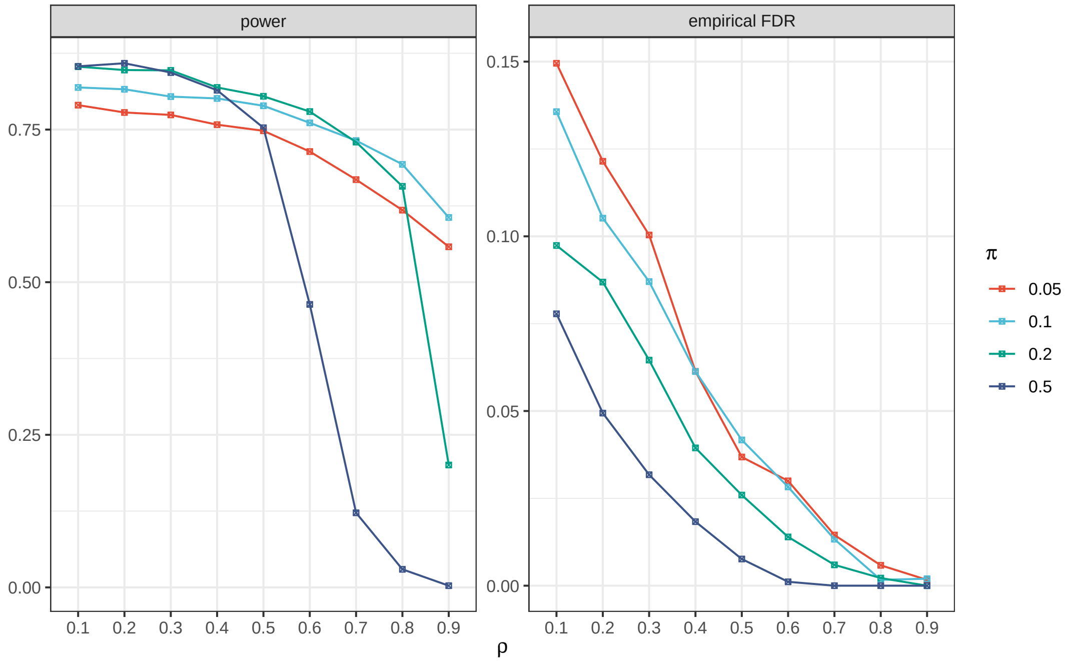

Figure S9: The average power and empirical FDR of mbDecoda on data simulated from the ZINB model, with ρ taking values in $\{0.1, 0.2, 0.3, 0.4, 0.5, 0.6, 0.7, 0.8, 0.9\}$, in which the variable of interest was binary and there were no confounders, with $\eta = 0.3$, and $\pi \in \{0.05, 0.1, 0.2, 0.5\}$.

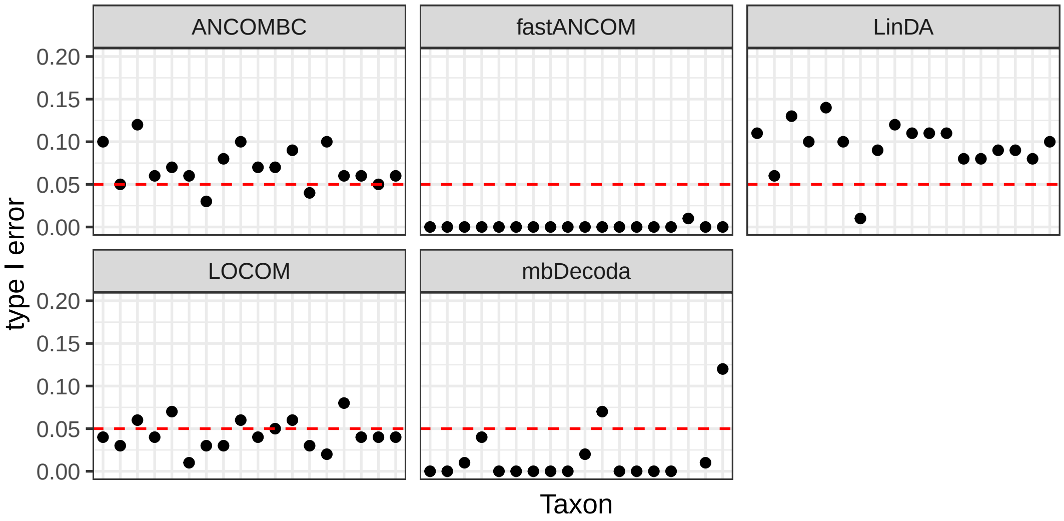

Figure S10: Type I error of various methods, without FDR control, on the HMP healthy cohort data. The 5% nominal level is indicated by a red dashed line.

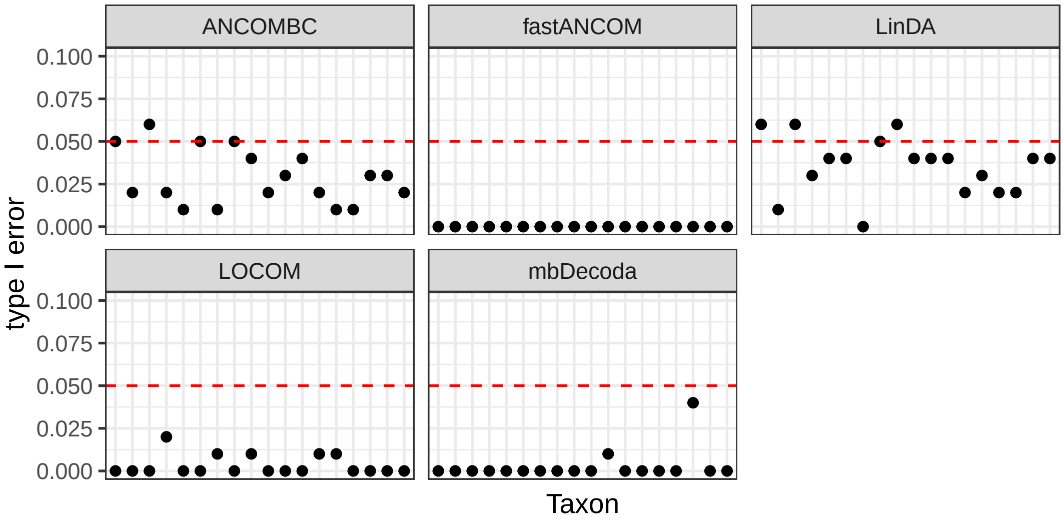

Figure S11: Type I error of various methods, with FDR control, on the HMP healthy cohort data. The 5% nominal level is indicated by a red dashed line.

Supplemental tables

Table S1: The average power and empirical FDR of various methods on additional data simulated from the ZINB model, in which the variable of interest was binary and there were no confounders, with $\pi = 0.2$, and $\eta = 0.3$.

		mbDecoda	ANCOMBC	fastANCOM	LinDA	LOCOM
$(n, K) = (50, 20)$	power(%)	79.00	18.50	10.00	10.50	65.50
	empirical FDR(%)	2.08	10.03	8.50	9.17	4.67
$(n, K) = (50, 50)$	power(%)	81.20	20.70	10.90	11.20	66.80
	empirical FDR(%)	3.15	14.08	14.42	7.90	4.49
$(n, K) = (100, 100)$	power(%)	90.65	37.35	14.75	27.10	86.25
	empirical FDR(%)	1.76	6.90	6.83	5.87	2.87
$(n, K) = (200, 100)$	power(%)	96.45	61.50	34.15	51.70	94.70
	empirical FDR(%)	2.38	5.31	1.50	6.26	3.20

Table S2: The average power and empirical FDR of various methods on data simulated from the ZINB model, in which the variable of interest was binary and there were no confounders, with $(n, K) = (50, 100)$, and $\pi = 0.2$. In this simulation, 50% of η_k equaled 0, while the remaining 50% were uniformly distributed between 0 and 0.7.

	mbDecoda	ANCOMBC	fastANCOM	LinDA	LOCOM
power(%)	83.15	65.60	43.35	61.05	77.45
empirical FDR(%)	2.62	6.43	4.63	4.51	3.89
